# Mechanisms of action of the hexane extract of *Hypericum brasiliense* and its component uliginosin B against drug-resistant *Staphylococcus aureus*

**DOI:** 10.5599/admet.3333

**Published:** 2026-07-06

**Authors:** Julia Chaves Scaffo, Sofia Trindade Mussi da Silva, Vitor Won-Held Rabelo, Leandro Stefano Sangenito, Lucas da Silva Abreu, Thaís P. Mello, Leandro Rocha, André Luis Souza dos Santos

**Affiliations:** 1 Graduate Program in Applied Sciences for Health Products, Faculty of Pharmacy, Fluminense Federal University (UFF), Niterói, Brazil; 2 Natural Products Technology Laboratory, Faculty of Pharmacy, Fluminense Federal University (UFF), Niterói, Brazil; 3 Laboratory of Advanced Studies of Emerging and Resistant Microorganisms, Paulo de Góes Microbiology Institute, Federal University of Rio de Janeiro (UFRJ), Rio de Janeiro, Brazil; 4 Laboratory of Chemistry of Natural Products, Institute of Chemistry, Fluminense Federal University (UFF), Niterói, Brazil; 5 Department of Virology, Paulo de Góes Microbiology Institute, Center of Health Science, Federal University of Rio de Janeiro (UFRJ), Rio de Janeiro, Brazil; 6 Collection of Environment and Health Fungi (CFAS), Fungal Section, Department of Microbiology, National Institute for Quality Control in Health (INCQS), Oswaldo Cruz Foundation (Fiocruz), Rio de Janeiro, RJ, Brazil

**Keywords:** Natural products, antimicrobial activity, bacterial infections, biofilm, toxicity, *Galleria mellonella*

## Abstract

**Background and purpose:**

Microbial resistance is a major global health concern. Methicillin-resistant Staphylococcus aureus is particularly relevant due to its clinical significance, virulence and adaptability. In the search for novel antimicrobial agents, natural products emerged as important sources of bioactive molecules. Previous studies have identified *Hypericum brasiliense* as a plant with promising antibacterial properties.

**Experimental approach:**

We evaluated the susceptibility, mechanisms of action and toxicity of *H. brasiliense* hexane extract (heHb) and the isolated compounds uliginosin B (uliB), isouliginosin B and japonicin A against reference and drug-resistant *S. aureus* isolates.

**Key results:**

The heHb and uliB demonstrated strong antibacterial activity, exhibiting MICs of 3.125 to 6.25 μg mL^-1^ and MBCs of 6.25 to 12.5 μg mL^-1^. Isouliginosin B displayed moderate activity (MIC = 12.5 μg mL^-1^; MBC ≤50 μg mL^-1^), whereas japonicin A was inactive. Transmission and scanning electron microscopy revealed pronounced ultrastructural alterations in bacterial cells following exposure to either heHb or uliB, and these exposures induced oxidative stress without compromising plasma membrane integrity. Conversely, the antioxidant N-acetylcysteine partially restored bacterial growth. Docking analysis suggested that uliB might act as a competitive substrate for the NADH-2 enzyme and cytochrome bd oxidase. Moreover, heHb and uliB inhibited biofilm formation and disrupted mature biofilms. Cytotoxicity assays (Vero and HaCaT lineages) showed CC₅₀ >90 μg mL^-1^, and haemolysis occurred only at the highest concentrations. *In vivo* assessment using *Galleria mellonella* confirmed low toxicity. ADMET predictions indicated a favourable pharmacokinetic profile for uliB.

**Conclusion:**

UliB is likely a key bioactive compound of *H. brasiliense*, with promising therapeutic potential against S. aureus.

## Introduction

*Staphylococcus aureus* is a Gram-positive bacterium that asymptomatically colonizes approximately 20 to 30 % of the global population [1]. Under conditions of dysbiosis or impaired host immunity, *S. aureus* can shift from a commensal to an opportunistic pathogen, causing a broad spectrum of infections ranging from mild skin and soft tissue lesions to severe, life-threatening systemic diseases [2]. Treatment of *S. aureus* infection has become increasingly challenging due to the rising prevalence of antimicrobial resistance [3], which has significantly reduced the effectiveness of available therapeutic options. This resistance is commonly driven by mutations at antimicrobial target sites [4] and is further compounded by *S. aureus*'s ability to form highly structured, resilient biofilms [1], which enhance tolerance to antimicrobial agents and host immune defences.

Natural products have long been recognized as prolific sources of novel therapeutic agents, playing a central role in the discovery and development of treatments for infectious diseases. Their remarkable chemical diversity and evolutionary optimization generate unique molecular scaffolds capable of targeting microbial pathogens through mechanisms often distinct from those of conventional drugs [5,6]. In plants, these bioactive metabolites serve as natural defence compounds, giving rise to a vast, chemically rich reservoir of molecules with antimicrobial potential [7,8]. In this context, *Hypericum brasiliense*, a species of the *Hypericaceae* family, has a well-documented history of medicinal use, attributed to its astringent, aromatic, excitant, vulnerary, antispasmodic and antiophidic properties. The apolar extract of *H. brasiliense* leaves and flowers contains a diverse array of bioactive metabolites, including phloroglucinols such as japonicin A, uliginosin A, uliginosin B, isouliginosin B and various hyperbrasilol derivatives [9-11]. Previous studies from our research group have demonstrated that the hexane extract, as well as the isolated compounds uliginosin B, isouliginosin B and japonicin A, display antibacterial activity against Gram-positive bacterial pathogens, including *Staphylococcus epidermidis* and *S. aureus* [12,13]. These findings underscore *H. brasiliense* as a promising source of natural antimicrobial agents. Nevertheless, the mechanisms underlying its antibacterial effects, particularly against drug-resistant *S. aureus* clinical isolates, remain insufficiently explored.

The present study aimed to advance current knowledge on the anti-staphylococcal activity of the hexane extract of *H. brasiliense* and its major phloroglucinol derivatives, including uliginosin B, isouliginosin B and japonicin A, against both reference strains and drug-resistant clinical isolates of *S. aureus*. Building upon previous findings, we comprehensively investigated their antimicrobial potential, including their ability to inhibit biofilm formation and disrupt established biofilms through assessments of metabolic activity, total biomass and extracellular matrix production. In addition, we explored potential mechanisms of action using transmission and scanning electron microscopies to visualize ultrastructural alterations in *S. aureus*. Complementary assays were performed to evaluate plasma membrane integrity, oxidative stress induction and changes in microbial metabolic activity. Finally, *in silico* molecular docking and pharmacokinetic analyses were conducted to test the hypothesis that uliginosin B interacts with and potentially disrupts components of the *S. aureus* respiratory chain, providing mechanistic insights into its antibacterial activity.

## Experimental

### Plant material

*Hypericum brasiliense* was collected in the municipality of Trajano de Moraes, Rio de Janeiro (22°12′17″ S, 43°11′35″ W), under authorization from SISBIO/ICMBio (no. 13659-21) and SisGen (A491A56). Botanical identification was performed by Dr. Marcelo Guerra (State University of Rio de Janeiro, UERJ), and voucher specimens were deposited in the Herbarium of the Faculty of Teacher Training (FFP/UERJ) to ensure traceability of the plant material. The whole plant was ground in a hammer mill, and the resulting powder was subjected to static maceration with hexane, following previously described procedures [11]. After extraction, the solvent was filtered and evaporated under reduced pressure using a rotary evaporator to obtain the crude extract. The extract was subsequently lyophilized and designated as the hexane extract of *H. brasiliense* (heHb), and the isolation of japonicin A, isouliginosin B and uliginosin B (uliB) was performed according to França *et al.* [9].

### Bacterial strain lineages

The bacterial panel used in this study included the methicillin-susceptible *S. aureus* (MSSA) reference strain ATCC 29213, obtained from the American Type Culture Collection (USA). Methicillin-resistant *S. aureus* (MRSA) strains, including USA300 and the Brazilian epidemic clone (BEC) HU25, were generously provided by Dr. Fabio Aguiar Alves (Fluminense Federal University, Brazil, and Palm Beach Atlantic University, USA). Additional clinical isolates (CR14-005, CR14-021, CR14-026 and CD16-016), as well as the BEC strain BMB 9393, were kindly supplied by Dr. Agnes Marie Sá Figueiredo/Dr. Bernadete Teixeira Ferreira Carvalho (Federal University of Rio de Janeiro, Brazil). These clinical strains were originally recovered from patients at the Clementino Fraga Filho University Hospital of UFRJ, Brazil [14]. Among the clinical isolates, CR14-005 exhibited resistance to clindamycin, erythromycin, chloramphenicol, ciprofloxacin and cefoxitin; CR14-021 showed resistance only to cefoxitin; CR14-026 was resistant to erythromycin, gentamicin and cefoxitin, with intermediate susceptibility to chloramphenicol; and CD16-016 presented resistance to clindamycin, erythromycin, chloramphenicol, ciprofloxacin and cefoxitin. All strains were stored in brain heart infusion (BHI) broth with 1 % glycerol at -20 °C.

### Evaluation of the antibacterial activity of H. brasiliense hexane extract and its purified compounds

For the antimicrobial susceptibility assays, serial dilutions of heHb and the isolated compounds uliB, isouliginosin B and japonicin A were prepared following CLSI M100-Ed33 guidelines in 96-well microtiter plates containing Mueller-Hinton broth, yielding final concentrations ranging from 100 to 0.78 μg mL^-1^. The bacterial inoculum was adjusted to a 0.5 McFarland standard (≈10^8^ colony-forming units (CFUs) mL mL^-1^) and subsequently diluted to obtain a final concentration of 10^4^ CFU mL^-1^ in each well. Plates were incubated for 24 h at 37 °C. Bacterial viability was assessed visually by inspecting the turbidity of each well, and the last well showing no visible growth was considered the minimum inhibitory concentration (MIC). To determine the minimum bactericidal concentration (MBC), 10 μL aliquots from wells of the MIC assay were plated onto Mueller-Hinton agar and incubated for 24 h at 37 °C. The MBC was defined as the lowest concentration that resulted in no visible bacterial growth [15].

### Assessment of the antibacterial activity of H. brasiliense hexane extract and uliginosin B under varying bacterial densities

To assess the inoculum effect on metabolic activity, bacterial suspensions with initial concentrations of 10^4^, 10⁵, 10⁶, 10^7^ or 10^8^ CFU mL^-1^ were cultured in 96-well plates with or without heHb and uliB at MIC values for 24 h at 37 °C. Following incubation, the 2,3-bis (2-methoxy-4-nitro-5-sulphenyl)-(2H)-tetrazolium-5-carboxanilide (XTT) and menadione solution were added to quantitatively assess cellular metabolic activity *via* tetrazolium reduction. The plates were further incubated for 3 h at 37 °C. Metabolic activity was finally quantified by measuring absorbance at 492 nm using a microplate reader Multiskan SkyHigh spectrophotometer (Thermo Fisher Scientific, Waltham, MA, USA).

### Effects of H. brasiliense hexane extract and uliginosin B on bacterial ultrastructure

Microscopy analyses were performed following the standardized procedures of the Microscopy Unit (UniMicro, UFRJ). *S. aureus* strains ATCC 29213 and USA300, previously cultured on tryptic soy agar (TSA), were inoculated into BHI broth, adjusted to 10^8^ CFU mL^-1^, and incubated in the absence or presence of heHb and uliB at their respective MIC values (6.25-3.125 μg mL^-1^) for 24 h at 37 °C. After incubation, supernatants were removed, and the cells were washed once with phosphate-buffered saline (PBS, pH 7.2). For scanning electron microscopy (SEM), bacterial samples were fixed for 1 h at room temperature in a solution containing 25 % glutaraldehyde, 0.2 M sodium cacodylate buffer, and Milli-Q water. Following three washes with 0.1 M sodium cacodylate buffer, cells were post-fixed with 1 % osmium tetroxide for 1 h and washed again with the same buffer. Dehydration was performed through a graded ethanol series (30, 50, 70, 90 and 100 %), followed by critical point drying with CO₂. Samples were then sputter-coated and examined using a Thermo Fisher Quattro S scanning electron microscope. For transmission electron microscopy (TEM), the protocol was followed up to the post-fixation step. Samples were subsequently dehydrated through a graded acetone series (30, 50, 70, 90 and 100 %), infiltrated with Spurr resin at room temperature, and polymerized at 68 °C for 72 h. Ultrathin sections were stained with uranyl acetate and alkaline lead citrate (5 to 10 min each) and visualized using a FEI Tecnai Spirit Bio-Twin transmission electron microscope.

### Effects of H. brasiliense hexane extract and uliginosin B on bacterial plasma membrane integrity

Bacteria grown overnight on TSA were adjusted to a final concentration of 10^7^ CFU mL mL^-1^ [16]. Cell suspensions were then incubated for 3 h in the presence or absence of heHb and uliB at concentrations ranging from 4×MIC to ½×MIC (25 to 1.56 μg mL mL^-1^). Boiled cells were included as a positive control for membrane disruption, untreated cells incubated with the probe served as a negative control, and unstained cells were used to assess autofluorescence. Following incubation, samples were centrifuged at 4,000 rpm for 10 min, washed once with PBS, and stained with propidium iodide (PI, 1 μg mL^-1^) for 10 min at 37 °C in the dark [17]. PI fluorescence was measured in the FL3 channel using a BD LSRFortessa™ flow cytometer, and at least 10,000 events were recorded per sample to ensure statistical robustness. Data were analyzed using Flowing Software version 2.5.1, and fluorescence intensity was visualized in side-scatter (SSC) versus PI plots to quantify the proportion of PI-positive cells, which indicate membrane-compromised populations.

### Effects of H. brasiliense hexane extract and uliginosin B on bacterial metabolic activity

Bacterial cells grown overnight on TSA were inoculated into BHI broth, adjusted to 10^7^ CFU mL mL^-1^, and exposed to heHb and uliB at concentrations ranging from 4×MIC to ½×MIC (25-1.56 μg mL^-1^) for 3 h at 37 °C. Untreated cells were used as the positive control [18]. Following incubation, supernatants were discarded, cells were washed once with PBS, and an XTT solution (200 μg mL^-1^) supplemented with menadione (0.4 mM) was added. Samples were then incubated for an additional 3 h at 37 °C. Metabolic activity was quantified by measuring absorbance at 492 nm using a Multiskan SkyHigh spectrophotometer (Thermo Fisher Scientific, Waltham, MA, USA).

### Effects of H. brasiliense hexane extract and uliginosin B on bacterial induction of reactive oxygen species

Bacterial cells grown overnight in TSA were adjusted to a 0.5 McFarland standard (≈10^8^ CFU mL^-1^) in BHI broth and exposed to the MIC concentrations of heHb and uliB (6.25 and 3.125 μg mL^-1^) for 30 min at 37 °C. The reactive oxygen species (ROS) assays used a 30 min incubation to capture early oxidative responses that would not be accurately represented at longer time points. After treatment, cells were incubated for an additional 30 min with 10 μM of the probe 2’,7’-dichlorodihydrofluorescein diacetate (H₂DCF-DA; Sigma-Aldrich, USA) [19]. Cells treated with hydrogen peroxide (50 mM) served as positive control, while untreated probe-incubated cells served as a negative control, and unlabelled cells were included to account for autofluorescence [20]. Fluorescence measurements were obtained using black 96-well plates (excitation 488 nm, emission 535 nm) on a SpectraMax M3 microplate reader (Molecular Devices). To normalize fluorescence signals for variations in bacterial density, cultures were subsequently transferred to transparent plates for absorbance measurements at 600 nm. In parallel, the effect of the antioxidant N-acetylcysteine (NAC) on bacterial growth (ATCC 29213) at the MIC was evaluated by measuring optical density at 600 nm (OD_600_). Bacterial suspensions prepared as described above were exposed to the previously determined MIC values of heHb and uliB in the presence or absence of NAC (final concentration of 5 mM) and incubated at 37 °C for 18-24 h. Following incubation, OD_600_ was measured using the Multiskan SkyHigh spectrophotometer (Thermo Fisher Scientific, Waltham, MA, USA) to assess bacterial growth. Comparisons between conditions with and without NAC were used to determine the influence of antioxidant supplementation on bacterial growth inhibition at the MIC level.

### Effects of H. brasiliense hexane extract and uliginosin B on bacterial biofilm formation and disruption

*S. aureus* was initially cultured on TSA for 24 h at 37 °C and subsequently transferred to TSB supplemented with 1 % glucose for an additional 24 h. The cultures were then adjusted to a final density of 10^8^ CFU mL mL^-1^. For biofilm formation assays, standardized bacterial suspensions were inoculated into 96-well polystyrene microtiter plates and incubated with heHb and uliB at concentrations ranging from 2×MIC to ¼×MIC (12.5 to 0.78 μg mL^-1^). Untreated cells served as positive controls. To evaluate biofilm disruption, mature 24 h pre-formed biofilms were exposed to higher compound concentrations, ranging from 8×MIC to MIC (50 to 3.125 μg mL^-1^), for an additional 24 h. In both experimental setups (biofilm formation and disruption), the well contents were gently aspirated, the wells were washed once with PBS, and the remaining biofilms were subsequently analysed for three classical parameters: biomass, metabolic activity (viability), and extracellular matrix (ECM) content. Biomass was quantified in methanol-fixed biofilms using crystal violet staining, with absorbance measured at 590 nm on a Multiskan SkyHigh spectrophotometer (Thermo Fisher Scientific, Waltham, MA, USA) [21,22]. ECM content was assessed in non-fixed biofilms at 530 nm following safranin incorporation [23,24]. Metabolic activity was evaluated in non-fixed biofilms using the XTT reduction assay (200 μg mL^-1^ XTT with 0.4 mM menadione), with absorbance measured at 492 nm [25].

### Effects of uliginosin B on bacterial oxidative phosphorylation proteins: a molecular docking approach

The three-dimensional structure of *S. aureus* NADH:quinone oxidoreductase type II (NDH-2) was retrieved from the Protein Data Bank (PDB ID: 5NA1). As no experimental structure is available for *S. aureus* cytochrome *bd* oxidase, a structural model of subunit I (CydA) predicted by AlphaFold2 (A0A2S6D6T4) was employed. Heme cofactors were incorporated *via* structural alignment with the E. coli cytochrome bd oxidase (PDB ID: 7OSE), followed by energy minimization in Swiss-PdbViewer 4.1. The resulting CydA model displayed satisfactory stereochemical quality, with 94.6 % of residues located in favoured regions of the Ramachandran plot. Ligand structures (uliB, quinestrol and menadione) were obtained from PubChem, geometry-optimized using OpenBabel 3.1.1, and subsequently energy-minimized under the MMFF94 force field. Partial atomic charges were assigned using the EEM method at the DFT-B3LYP/6-311G/NPA level. Molecular docking simulations were performed using AutoDock Vina 1.1.2, treating protein structures as rigid and ligands as fully flexible. Docking grids were cantered on previously characterized quinone/quinol binding sites. Resulting protein-ligand complexes were visualized and analysed using PyMOL and Discovery Studio Visualizer 2021 (Dassault Systèmes BIOVIA, San Diego, CA).

### Effects of H. brasiliense hexane extract and uliginosin B on the toxicity of mammalian cell lineages

The HaCaT (human keratinocyte) and Vero (monkey kidney epithelial) cell lines were maintained in Dulbecco's Modified Eagle Medium (DMEM) supplemented with 10 % foetal bovine serum (FBS) at 37 °C in a 5 % CO₂ atmosphere. Mammalian cells (10⁵ cells *per* well) were first seeded in 96-well tissue culture plates and allowed to adhere for 4 h under standard culture conditions. Non-adherent cells were then removed by gentle washing with sterile DMEM, after which the wells were replenished with fresh DMEM containing 10 % FBS. Cells were exposed to increasing concentrations of heHb and uliB (7.81-500 μg mL^-1^) and incubated for an additional 24 h at 37 °C in a 5 % CO₂ atmosphere. Following treatment, the culture medium was discarded and 3-(4,5-dimethylthiazol-2-yl)-2,5-diphenyltetrazolium bromide (MTT) was added to each well (25 μg *per* well). Plates were incubated for 3 h in the dark at 37 °C, centrifuged at 500 × g for 8 min, and the supernatant removed. The resulting formazan crystals were dissolved in 200 μL of DMSO, and absorbance was measured at 570 nm using a SpectraMax M3 microplate reader (Molecular Devices). The 50 % cytotoxic concentration (CC₅₀) was calculated by nonlinear regression analysis [26].

### Effects of H. brasiliense hexane extract and uliginosin B on erythrocyte lysis

Two-percent sheep erythrocytes (Cultilab, Rio de Janeiro, RJ) were incubated in 96-well plates with serial dilutions of heHb and uliB, starting at 100× MIC (625-2.44 μg mL^-1^), for 3 or 24 h at 37 °C. Following incubation, the plates were centrifuged at 2,100 rpm for 10 min, and the supernatants were collected for absorbance measurement at 415 nm using a Multiskan SkyHigh spectrophotometer (Thermo Fisher Scientific, Waltham, MA, USA) [27]. Triton X-100 (0.1 %) was used as the positive control for complete haemolysis, whereas PBS served as the negative control.

### Effects of H. brasiliense hexane extract and uliginosin B on Galleria mellonella larval survivability

*Galleria mellonella* larvae weighing approximately 0.2-0.3 g and displaying clear, uniform coloration were selected for the *in vivo* toxicity assay. Experimental groups consisted of 10 larvae each, including a negative control group injected with PBS. The compounds heHb (625 μg mL^-1^) and uliB (312.5 μg mL^-1^), corresponding to 100× MIC, were tested by injecting 10 μL of each solution into the last left proleg using an insulin syringe (BD Ultra-Fine, Franklin Lakes, NJ, USA). Following injection, the larvae were incubated at 37 °C for 168 h. Larval survival was monitored daily. Death was determined by the absence of movement in response to gentle physical stimuli applied to the head and body, as well as by the presence of extensive melanisation. Throughout the entire experimental period, all larvae were maintained under controlled conditions at 37 °C.

### Determination of the selectivity index for H. brasiliense hexane extract and uliginosin B

The selectivity index (SI) of heHb and uliB was determined as the ratio between their MIC values against *S. aureus* and their CC₅₀ values in mammalian cells and in *G. mellonella* larvae. Compounds exhibiting SI values greater than 10 were considered promising candidates for further investigation [28].

### In silico analysis of pharmacokinetic, toxicological and drug-like properties of uliginosin B

The pharmacokinetic, toxicological, and drug-likeness properties of uliB were predicted using computational approaches, with vancomycin as the reference compound. Human intestinal absorption, blood-brain barrier permeability and P-glycoprotein interactions were evaluated using the admetSAR 3 server [29]. The Deep-PK platform was used to predict potential substrate or inhibitory activity toward major CYP450 isoforms, including CYP1A2, CYP2C9, CYP2C19, CYP2D6 and CYP3A4 [30]. Drug-likeness was assessed according to Lipinski’s “Rule of Five” and the Pfizer 3/75 rule, which provide estimates of oral bioavailability and preclinical toxicity risk [31]. Predicted toxicological endpoints, including genotoxicity, carcinogenicity, hepatotoxicity, nephrotoxicity, cardiotoxicity (hERG inhibition), respiratory toxicity and skin or eye irritation, were also evaluated through admetSAR 3.

### Statistical analysis

All experiments were performed at least three times in biological triplicate. Triplicate values for each sample were averaged, and results were analysed by one-way ANOVA with a 95 % confidence level (*p* < 0.05), using Dunnett's post hoc test to compare treated samples with untreated controls or Sidak for multiple comparisons. *In vitro* toxicity data were analysed using Student’s t-test with a 95% confidence level (*p* < 0.05). *In vivo* toxicity was assessed using the Mantel-Cox log-rank test. All statistical analyses were performed using GraphPad Prism 8.0.1 [25].

## Results and discussion

### Inhibitory effects of H. brasiliense hexane extract and purified compounds on the planktonic growth of S. aureus

In this experimental set, a panel of eight *S. aureus* strains representing both MSSA and MRSA phenotypes was evaluated. In this context, *S. aureus* ATCC 29213 was included as the reference MSSA strain, while the MRSA group comprised two Brazilian epidemic clones (BECs HU25 and BMB9393), the community-associated USA300 lineage, and four clinical isolates (CR14-005, CR14-021, CR14-026 and CD16-016). All bacterial strains were tested against heHb and its purified components using the broth microdilution method in accordance with CLSI guidelines. Both heHb and uliB exhibited comparable inhibitory and bactericidal activities across the diverse genetic backgrounds analysed, with MIC values ranging from 3.125 to 6.25 μg mL^-1^ and MBC values from 6.25 to 12.5 μg mL^-1^ (Table 1). This consistency across MSSA and MRSA strains highlights the broad-spectrum and robust antibacterial potential of these compounds. Additionally, the close correspondence between MIC and MBC values indicates that heHb and uliB act predominantly through a bactericidal mechanism. Among the remaining isolated metabolites, isouliginosin B showed only moderate antibacterial activity, with MIC values of 6.25-12.5 μg mL^-1^ and MBC values of 12.5-50 μg mL^-1^, while japonicin A exhibited no detectable activity (MIC >100 μg mL^-1^) (Table 1). Geometric mean values were calculated for all strains and are presented in Table 1. Collectively, these findings further emphasize uliB as a bioactive component responsible for the potent anti-*S. aureus* effects observed in heHb. Given their pronounced efficacy, both heHb and uliB were selected for subsequent experimental analyses to elucidate their mechanisms of action and biological effects.

**Table 1.** Minimum inhibitory (MIC) and bactericidal (MBC) concentrations of the hexane extract of *H. brasiliense* and its isolated compounds against diverse *S. aureus* strains.

*S. aureus* strains	Concentration, μg mL^-1^							
	heHb		uliB		Isouliginosin B		Japonicin A	
	MIC	MBC	MIC	MBC	MIC	MBC	MIC	MBC
ATCC 29213	6.25	12.5	3.125	6.25	12.5	25	>100	>100
HU 25	3.125	6.25	3.125	3.125	12.5	25	>100	>100
USA 300	6.25	12.5	3.125	6.25	12.5	25	>100	>100
BMB 9393	6.25	6.25	3.125	6.25	12.5	25	>100	>100
CR14-005	3.125	6.25	3.125	6.25	12.5	50	>100	>100
CR14-021	3.125	6.25	6.25	12.5	6.25	25	>100	>100
CR14-026	3.125	6.25	3.125	6.25	6.25	12.5	>100	>100
CD16-016	6.25	12.5	3.125	12.5	12.5	25	>100	>100
Geometric mean	4.42	8.11	3.41	6.82	10.51	25.00	>100	>100

### Influence of inoculum size on the activity of H. brasiliense hexane extract and uliginosin B against S. aureus

**Figure 1.**
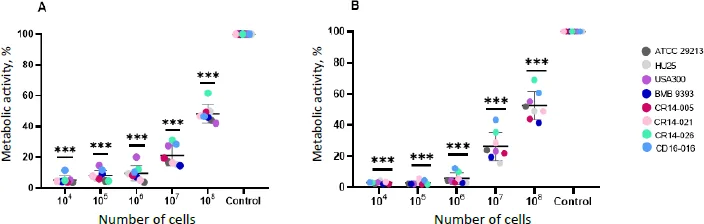
Inoculum effect and metabolic activity of *S. aureus* treated with heHb and uliB. Bacterial suspensions ranging from 10^4^ to 10^8^ cells were incubated for 24 h at 37 °C with MIC concentrations of heHb (6.25 μg mL^-1^) (A) or uliB (3.125 μg mL^-1^) (B). Untreated cells were used as the control. Metabolic activity was assessed using the XTT assay based on tetrazolium salt reduction by metabolically active cells. Statistical analyses were performed using one-way ANOVA followed by Dunnett’s post hoc test, with a significance level of 95 % (*p* <0.05) and Dunnett's post-test. ***p <0.0001

Given the promising inhibitory effects of heHb and uliB on the proliferation of *S. aureus*, we next investigated whether these compounds were subject to an inoculum-dependent response. To this end, heHb and uliB were tested at their respective MICs against a range of initial bacterial densities (10^4^, 10⁵, 10⁶, 10^7^ and 10^8^ CFU mL^-1^) and incubated for 24 h. Both compounds maintained strong activity at lower inoculum levels (10^4^ to 10⁶ CFU mL^-1^), reducing metabolic activity by more than 80 %. Although a diminished response was observed at the highest bacterial loads (10^7^ to 10^8^ CFU mL^-1^), metabolic activity was still reduced by approximately 50 % (Figure 1). These results indicate that heHb and uliB retain substantial inhibitory activity even under elevated inoculum conditions, supporting their robustness and potential applicability against *S. aureus* populations of varying densities.

### Alterations in the ultrastructure of S. aureus induced by H. brasiliense hexane extract and uliginosin B

To investigate the effects of heHb and uliB on the ultrastructure of *S. aureus* cells, SEM and TEM analyses were conducted. Two representative strains were selected: USA300 (MRSA), due to its global dissemination and clinical relevance, and ATCC 29213 (MSSA), which served as a standard reference strain. Bacterial cells were exposed to heHb and uliB at their respective MIC concentrations for 24 h prior to imaging.

**Figure 2.**
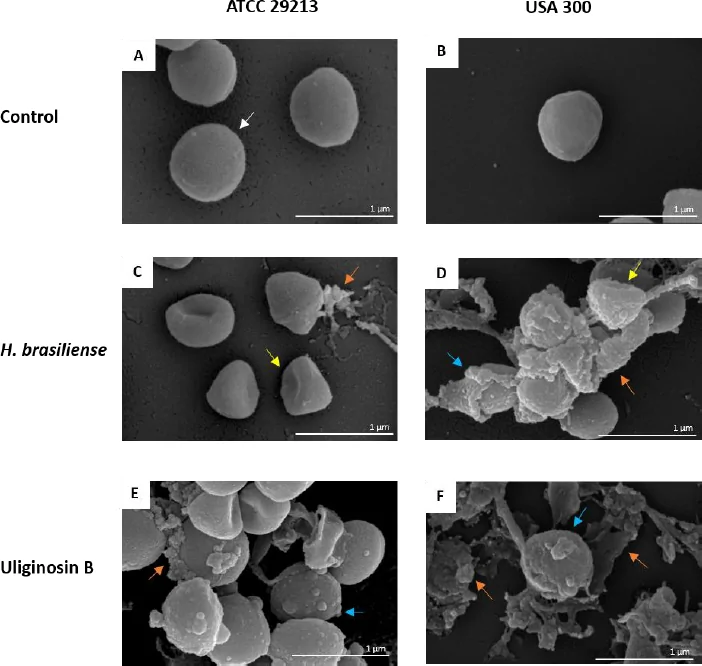
Scanning electron microscopy of *S. aureus* ATCC 29213 and USA 300 treated with heHb and uliB for 24 h. A and B - untreated *S. aureus* cells. C and D - Cells treated with heHb at the MIC concentration (6.25 or 3.13 μg mL^-1^). E and F - cells treated with uliB at the MIC concentration (6.25 or 3.13 μg/mL). White arrows indicate intact, well-defined cocci; yellow arrows indicate cell surface depression; orange arrows indicate cell lysis and extravasation of intracellular contents. Blue arrows indicate vesicles in the cell wall.

SEM analyses revealed clear ultrastructural differences between untreated and treated *S. aureus* cells. Untreated MSSA ATCC 29213 and MRSA USA300 cells (Figs. 2A and 2B) displayed smooth surfaces, intact cell walls and well-preserved spherical morphology, with no signs of distortion or damage. In contrast, exposure to heHb (Figures 2C and 2D) induced marked morphological alterations, including pronounced surface depressions, membrane collapse, leakage of intracellular material, increased surface roughness, overproduction of extracellular vesicles and extensive cell lysis. Treatment with uliB (Figures 2E and 2F) produced a distinct ultrastructural profile compared with the crude extract. Bacterial cells exhibited severe membrane disruption, abundant vesicle formation, accumulation of cellular debris and multiple nodular or blister-like protrusions, particularly evident in the USA300 strain, indicating substantial membrane destabilization. Overall, the SEM findings demonstrate that both heHb and uliB compromise *S. aureus* cell integrity, with uliB inducing more pronounced membrane-focused damage.

**Figure 3.**
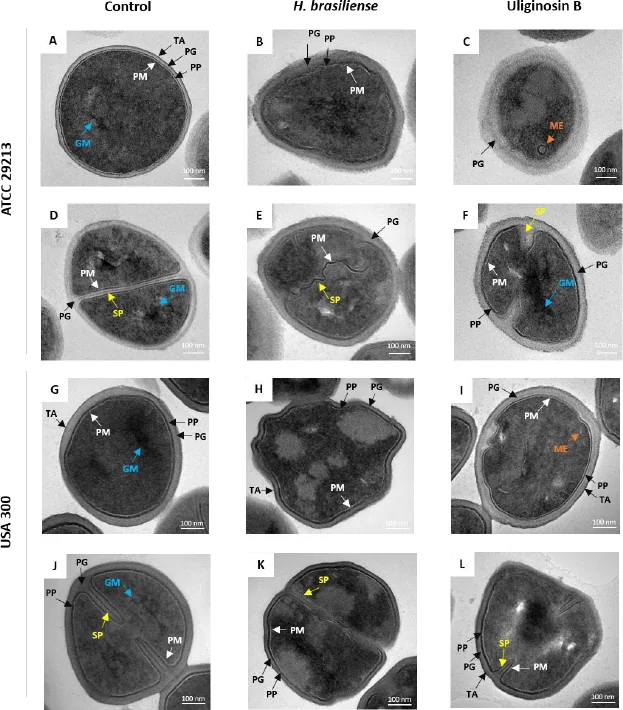
Transmission electron microscopy of *S. aureus* ATCC 29213 and USA 300 treated with heHb and uliB for 24 h. A /D and G/J - untreated ATCC 29213/ USA 300 cells; B/E and H/K - ATCC 29213/ USA 300 cells treated with heHb at MIC concentration (6.25 or 3.13 μg mL^-1^); C/F and I/L - ATCC 29213/ USA 300 cells treated with uliB at MIC concentration (6.25 or 3.13 μg mL^-1^). The black arrows indicate the cell wall with teichoic acids (TA); peptidoglycan (PG); periplasm (PP). White arrows indicate the plasma membrane (PM). Yellow arrows indicate the septum (SP). Blue arrows indicate the genetic material (GM). Orange arrows indicate mesosome-like structures (ME).

TEM analysis revealed that untreated cells of *S. aureus* ATCC 29213 and USA300 displayed well-preserved ultrastructure, characterized by homogeneous cytoplasmic density, intact plasma membranes and regular cell wall thickness (Figure 3A, 3C, 3G and 3J).

In contrast, bacterial cells exposed to heHb exhibited extensive structural disruption. In ATCC 29213, treated cells assumed amorphous shapes and showed clear defects in cell division, including impaired formation of the characteristic asymmetrical septum (Figures 3B and 3E). Similar abnormalities were observed in USA300, where heHb caused pronounced cytoplasmic disorganization, areas of reduced electron density indicative of intracellular degradation, thinning of the cell wall and severe membrane distortion (Figures 3H and 3K). Septum formation was irregular and incomplete, highlighting the extract’s disruptive impact on cell division machinery (Figure 3K). Treatment with uliB induced ultrastructural alterations broadly comparable to those caused by heHb; however, several distinctive features were observed.

UliB-treated cells exhibited increased and heterogeneous peptidoglycan thickness, highly irregular septum architecture and pronounced asymmetry during cell division. Additionally, signs of uneven chromosomal segregation and abnormal nucleoid distribution were evident (Figures 3C, 3F, 3I and 3L).

### Elucidating the mechanisms of action of H. brasiliense hexane extract and uliginosin B in S. aureus

**Figure 4.**
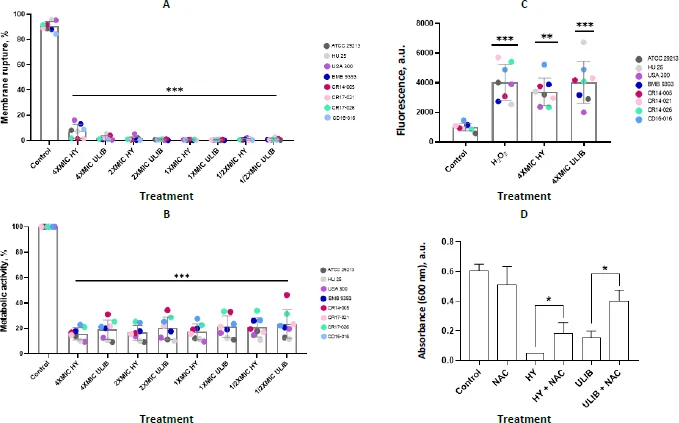
Mechanisms of action of heHb and uliB in *S. aureus*. (A) Plasma membrane disruption in *S. aureus* treated with heHb and uliB for 3 h at 4× to 1/2× MIC concentrations (25 to 1.56 μg mL^-1^). Heat-killed cells were used as the positive control, and untreated cells as the negative control. Membrane integrity was assessed using propidium iodide, a membrane-impermeable fluorescent probe that penetrates cells with compromised membranes and binds to nucleic acids. (B) Metabolic activity of *S. aureus* during treatment with heHb and uliB. Bacteria were cultured for 3 h in the presence or absence of heHb and uliB at 4× to 1/2× MIC concentrations (25 to 1.56 μg mL^-1^), with untreated cells used as the control. Metabolic activity was evaluated using the XTT assay, based on tetrazolium salt reduction by metabolically active cells. (C) Induction of reactive oxygen species (ROS) in *S. aureus* treated with heHb and uliB at 4× MIC concentrations (25 and 12.5 μg mL^-1^) for 30 min. ROS levels were detected using the H₂DCF-DA probe, which is converted intracellularly into a fluorescent compound upon oxidation by ROS. Cells treated with H₂O₂ were used as the positive control, and untreated cells as the negative control. (D) Effect of the antioxidant NAC on bacterial growth at the MIC of heHb and uliB. *S. aureus* (ATCC 29213) cells were exposed to the MIC values of each compound in the presence or absence of NAC, and bacterial growth was assessed by measuring OD_600_ after incubation. Statistical analyses were performed using one-way ANOVA followed by Dunnett’s post hoc test, with a significance level of 95% (*p* <0.05). *** < 0.0001; **p < 0.001.

To capture early cellular responses prior to extensive growth-related effects, planktonic cultures of *S. aureus* treated with heHb and uliB for 3 h were evaluated for plasma membrane integrity and metabolic activity. Membrane permeability was assessed using PI staining, whereas metabolic activity was quantified using the XTT assay. Treatment with heHb or uliB did not significantly compromise membrane integrity, as PI uptake remained comparable to that of untreated cells and markedly lower than the high PI incorporation observed in boiled cells, which served as the positive control for membrane disruption (Figure 4A). In contrast, both compounds induced a marked reduction in metabolic activity, decreasing XTT reduction by approximately 80 % across all tested concentrations. These findings indicate that heHb and uliB primarily impair cellular metabolism rather than causing overt membrane rupture (Figure 4B). To further explore their mechanisms of action, intracellular ROS levels were quantified. After 30 min of exposure to 4×MIC of heHb or uliB, a substantial increase in ROS production was detected with the H₂DCF-DA probe (Figure 4C). Statistical comparisons showed no significant differences between the treated groups and the positive control (H₂O₂), while untreated cells exhibited minimal fluorescence (Figure 4C). Collectively, these results suggest that oxidative stress plays a key role in the antibacterial activity of both heHb and uliB compounds.

Treatment of *S. aureus* cells with heHb and uliB at their respective MIC values resulted in a marked reduction in bacterial growth, as expected (Figure 4D). However, co-treatment with the antioxidant NAC partially restored bacterial growth, as evidenced by increased OD_600_ relative to treatments without NAC. This finding suggests that the antimicrobial activity of both compounds is at least in part associated with oxidative stress induction. Notably, the incomplete recovery of growth in the presence of NAC indicates that additional ROS-independent mechanisms also contribute to the bactericidal effects of heHb and uliB.

### Evaluation of the antibiofilm activity of H. brasiliense hexane extract and uliginosin B

**Figure 5.**
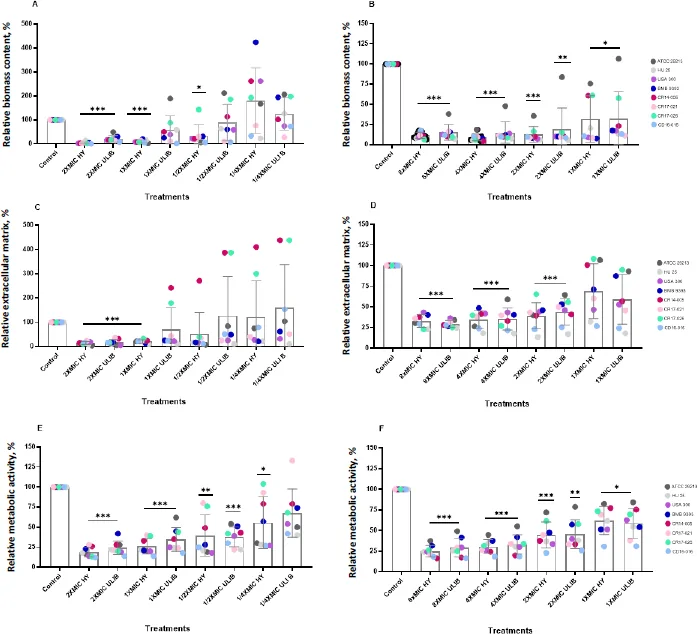
Antibiofilm activity of heHb and uliB against different strains of *S. aureus*. Biofilm formation was evaluated during treatment with heHb and uliB at concentrations ranging from 2× to 1/4× MIC (12.5 to 0.78 μg mL^-1^) by analysing total biomass using crystal violet (A), extracellular matrix production using safranin (C), and metabolic activity using the XTT assay (E). The effects on preformed mature biofilms were assessed at concentrations ranging from 8× - 1× MIC (50 to 3.125 μg mL^-1^) by measuring total biomass with crystal violet (B), extracellular matrix production with safranin (D) and metabolic activity with XTT (F). Statistical analyses were performed using one-way ANOVA followed by Dunnett’s post hoc test, with a significance level of 95 % (*p* <0.05). ***p 0,0001; **p < 0.0009; * *p* < 0.01

The effects of heHb and uliB on *S. aureus* biofilms were also investigated. For the biofilm formation assay, bacterial cultures were incubated for 24 h in the presence of increasing concentrations of heHb and uliB. Three classical biofilm parameters were quantified: total biomass, ECM and metabolic activity. The inhibition of biofilm formation was clearly concentration-dependent. At the highest concentration tested (2×MIC), both heHb and uliB reduced total biomass, ECM production and metabolic activity by more than 50 % (Figures 5A, 5C and 5E). Treatment with heHb at MIC produced inhibitory effects comparable to those observed at 2×MIC. In contrast, uliB at MIC exhibited strain-dependent behaviour, with some isolates still forming biofilms at levels similar to the untreated controls (Figs. 5A and 5B). Interestingly, exposure to sub-inhibitory concentrations (½×MIC and ¼×MIC) of both compounds led to a paradoxical increase in biofilm development, enhancing biomass, ECM content and metabolic activity beyond the levels observed in untreated controls (Figures 5A, 5C and 5E). This stimulatory effect at low concentrations suggests a stress-induced compensatory response commonly reported for *S. aureus* under sublethal antimicrobial pressure. The ability of heHb and uliB to disrupt mature *S. aureus* biofilms was also evaluated.

Across all tested concentrations, both the extract and the isolated compound significantly reduced total biomass, ECM production and metabolic activity compared with untreated controls (Figures 5B, 5D and 5F). At the highest concentrations (8×MIC, 4×MIC and 2×MIC), biofilm disruption reached approximately 50 to 90 % across several strains, demonstrating strong antibiofilm activity. Treatments at MIC produced strain-dependent responses but still achieved notable reductions, ranging from 25 to 80 %. Overall, these findings indicate that heHb and uliB are effective not only in preventing biofilm formation but also in impairing established biofilms, underscoring their broad antibiofilm potential against *S. aureus*.

### In silico prediction of uliginosin B molecular targets in S. aureus

Based on the mechanistic assays, both heHb and uliB primarily target the metabolic machinery of *S. aureus*, resulting in a marked reduction in metabolic activity, loss of cell viability and profound, irreversible damage to cellular morphology. Notably, this metabolic impairment was also evident in cells embedded within biofilms, indicating that the compounds effectively compromise bacterial activity in both planktonic and sessile states. These findings reinforce the idea that heHb and uliB act through metabolism-cantered mechanisms rather than through direct membrane disruption. To gain further insight into the molecular basis of this inhibitory effect, *in silico* analyses were performed to evaluate the binding potential of uliB to key enzymes of the *S. aureus* electron transport chain, particularly NADH:quinone oxidoreductase type II (NDH-2) and cytochrome *bd* oxidase. These enzymes are essential for maintaining cellular respiration and redox homeostasis, and their inhibition could plausibly explain the pronounced metabolic collapse observed *in vitro*.

**Figure 6.**
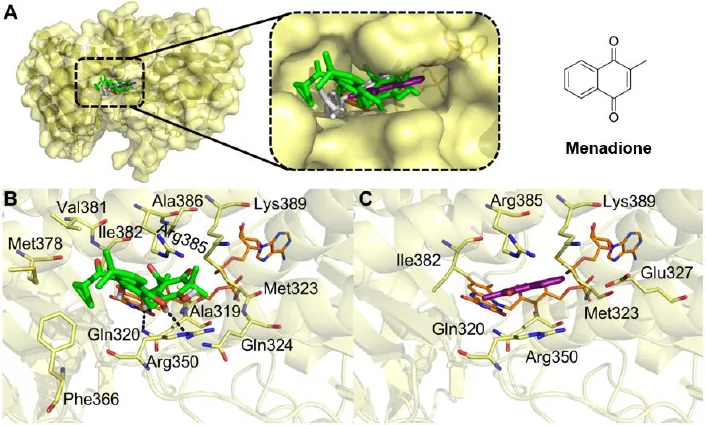
Molecular docking of uliB and the substrate menadione in the NDH-2 enzyme from *S. aureus*. (A) overlay of the predicted poses for uliB (green) and menadione (purple) complexed with the enzyme from *S. aureus* and ubiquinone (white) complexed with the homologous enzyme Ndi1 from *S. cerevisiae* (PDB code 4G73). Intermolecular interactions of (B) uliB and (C) menadione with the *S. aureus* enzyme. Hydrogen bonds are shown as black dashed lines. The FAD cofactor is shown in orange

Molecular docking analyses showed that uliB binds within the quinone-binding pocket of NDH-2, adopting an orientation that overlaps with that of the natural substrate, menadione (Figure 6). In the reference complex, menadione interacts with key residues of the quinone-binding site, including Gln_320_, Arg_350_, and Arg385, as well as Met323, Glu327, Ile382, and Lys389, through van der Waals contacts. UliB occupies the same core region, with one of its aromatic rings forming hydrogen bonds with Gln_320_ and Arg_350_, in addition to engaging in van der Waals interactions with Ala_319_, Met_323_, Ile_382_, Arg_385_, Ala_386_ and Lys_389_.

Due to its larger and more complex structure, uliB extends further toward the entrance of the binding pocket, where its second aromatic ring establishes additional stabilizing contacts with Gln_324_, Phe_366_, Met_378_ and Val_381_ (Figure 6). The predicted binding affinity of uliB (-25.104 kJ mol^-1^ (1 kJ = 0.239 kcal) was similar to that of menadione (-27.614 kJ mol^-1^), suggesting that uliB may competitively interfere with quinone binding and consequently disrupt electron transport, providing a plausible molecular explanation for the observed reduction in bacterial metabolic activity.

**Figure 7.**
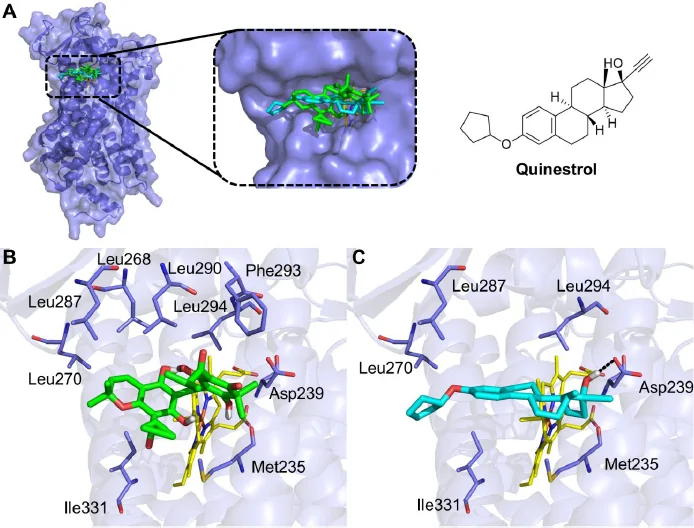
Molecular docking of uliB with cytochrome bd oxidase subunit I (CydA) from *S. aureus* and comparison with the steroidal inhibitor quinestrol. (A) overlap of the predicted binding mode for quinestrol (cyan) and uliginosin B (green). Intermolecular interactions between the protein and (B) uliB (green) or (C) quinestrol (cyan) are shown. Hydrogen bonds are shown as black dashed lines. The closest heme B group is shown in yellow

In subunit I of *S. aureus* cytochrome *bd* oxidase (CydA), uliB adopted a binding pose that overlapped substantially with that of the reference inhibitor quinestrol, resulting in a comparable interaction pattern (Figure 7). Owing to the predominantly hydrophobic characteristics of the binding pocket, van der Waals forces were the primary contributors to ligand stabilization. Both uliB and quinestrol engaged residues Met_235_, Leu_270_, Leu_287_, Leu_294_ and Ile_331_, which form the hydrophobic core of the pocket. In addition to these contacts, quinestrol established a hydrogen bond with Asp_239_, providing an extra stabilizing interaction absent in the uliB complex (Figure 7). Despite the similarity in binding orientation, quinestrol displayed a more favourable predicted binding energy (-29.29 kJ mol^-1^) relative to uliB (-20.92 kJ mol^-1^), indicating a higher theoretical affinity for CydA. This suggests that, although uliB can interact with cytochrome *bd* oxidase, its inhibitory potential for this enzyme may be lower than that inferred for quinestrol.

### Evaluation of the in vitro and in vivo toxicity profiles of H. brasiliense hexane extract and uliginosin B

Toxicity analyses were conducted to assess the safety of heHb and uliB across multiple experimental models. Both compounds exhibited low haemolytic activity, with ≤20 % haemolysis observed at the highest concentrations tested (156.2 μg mL^-1^ for heHb and 78.12 μg mL^-1^ for uliB), which correspond to 10 to 20× their MIC values. Because haemolysis did not reach 50 % at any tested concentration, the CC₅₀ for red blood cells could not be determined and is therefore inferred to be greater than these maximum values. Cytotoxicity toward mammalian cells was evaluated using Vero (kidney epithelial) and HaCaT (keratinocyte) cell lines. In Vero cells, the CC₅₀ values were 414.75 μg mL^-1^ for heHb and 97.6 μg mL^-1^ for uliB, whereas in HaCaT cells, the CC₅₀ values were 393.57 and 212.26 μg mL^-1^, respectively. These findings demonstrate that both compounds exhibit markedly greater selectivity for *S. aureus* than for mammalian cells. *In vivo* toxicity was assessed using *G. mellonella* larvae exposed to 100× MIC concentrations (625 μg mL^-1^ for heHb and 312.5 μg mL^-1^ for uliB). Larval mortality remained below 20 % throughout the 168 h observation period, with only one death recorded for heHb within 24 h and one for uliB within 72 h. No statistically significant differences were observed compared with the PBS control group, indicating good tolerability in the invertebrate model.

Based on these toxicity results, the SI for *S. aureus* was calculated using MIC and CC₅₀ values obtained from Vero and HaCaT cell lines (Table 2). All SI values exceeded 10, demonstrating a strong preferential activity of both compounds toward bacteria rather than mammalian cells. For heHb, SI values ranged from 66.4 to 132.9, whereas for uliB they ranged from 15.6 to 68. Because CC₅₀ values could not be determined for haemolysis or *G. mellonella* due to the minimal toxicity observed, the SI for these assays was summarized in Table 2, further reinforcing the high bacterial selectivity of heHb and uliB.

**Table 2.** Selectivity index of heHb and uliB with respect to *S. aureus* strains and Vero and HaCaT cells

*S. aureus* strains	Selectivity index							
	heHb				uliB			
	Vero	HaCaT	Erythrocytes	*G. mellonella*	Vero	HaCaT	Erythrocytes	*G. mellonella*
ATCC 29213	66.4	62.98	>100	>100	31.25	68	>100	>100
HU 25	132.9	126.14	>200	>200	31.25	68	>100	>100
USA300	66.4	62.98	>100	>100	31.25	68	>100	>100
BMB 9393	66.4	62.98	>100	>100	31.25	68	>100	>100
CR14-005	132.9	126.14	>200	>200	31.25	68	>100	>100
CR14-021	132.9	126.14	>200	>200	15.6	34	>50	>50
CR14-026	132.9	126.14	>200	>200	31.25	68	>100	>100
CD16-016	66.4	62.98	>100	>100	31.25	68	>100	>100

### Predicted pharmacokinetic, toxicological and drug-like properties of uliginosin B

To further characterize the pharmacokinetic and toxicological profile of uliB, *in silico* ADMET analyses were performed and compared with those of the reference antibiotic vancomycin. These assessments provide additional insight into the drug-likeness and potential safety of uliB as a therapeutic candidate. UliB demonstrated favourable pharmacokinetic attributes, including good human intestinal absorption (Table 3). It also complied with Lipinski’s “Rule of Five” [31], indicating a high likelihood of good oral bioavailability; unlike vancomycin, which, despite its clinical efficacy, is not orally bioavailable. Similar to vancomycin, uliB was not predicted to cross the blood-brain barrier under physiological conditions. With respect to P-glycoprotein (PgP), uliB was predicted to act as an inhibitor but not as a substrate (Table 3), a characteristic that may enhance its potential for synergistic interactions when combined with other antimicrobials. Toxicity predictions indicated that uliB is non-genotoxic, non-carcinogenic, and non-irritant to the skin and eyes, paralleling the profile of vancomycin. However, uliB showed positive predictions for hepatotoxicity and respiratory toxicity, whereas vancomycin was predicted to exhibit only respiratory toxicity.

**Table 3.** Predicted pharmacokinetic, toxicological and drug-like profile for the natural product uliginosin B and the antibiotic drug vancomycin, using the admetSAR 3 and Deep-PK servers. All models employed are classification-based and provide qualitative results for their respective endpoints. For the pharmaceutical rules (Lipinski’s “rule of five” and the Pfizer 3/75), the results indicate whether the compounds meet the criteria established by each rule (approved or rejected), as described in the text. HIA: human intestinal absorption; BBB: ability to cross the blood-brain barrier; PgP: P-glycoprotein

Predicted endpoint	Uliginosin B	Vancomycin
HIA	Absorbed	Non-Absorbed
Lipinski rule of five	Approved	Rejected
BBB	Non-Penetrable	Non-Penetrable
PgP substrate	Non-Substrate	Non-Substrate
PgP inhibitor	Inhibitor	Non-Inhibitor
CYP1A2 substrate	Substrate	Non-Substrate
CYP2C9 substrate	Non-Substrate	Non-Substrate
CYP2C19 substrate	Substrate	Non-Substrate
CYP2D6 substrate	Non-Substrate	Non-Substrate
CYP3A4 substrate	Substrate	Non-Substrate
CYP1A2 inhibitor	Inhibitor	Non-Inhibitor
CYP2C9 inhibitor	Non-Inhibitor	Non-Inhibitor
CYP2C19 inhibitor	Non-Inhibitor	Non-Inhibitor
CYP2D6 inhibitor	Non-Inhibitor	Non-Inhibitor
CYP3A4 inhibitor	Inhibitor	Non-Inhibitor
Pfizer rule 3/75	Approved	Approved
Genotoxicity	Low risk	Low risk
Carcinogenicity	Low risk	Low risk
Nephrotoxicity	Low risk	Low risk
Cardiotoxicity	Low risk	Low risk
Hepatotoxicity	High risk	Low risk
Skin irritation	Low risk	Low risk
Eye irritation	Low risk	Low risk
Respiratory toxicity	High risk	High risk

## Discussion

The increasing prevalence of antimicrobial resistance continues to outpace the development of effective therapeutics, reinforcing the need for new compounds capable of overcoming resistance mechanisms in *S. aureus* [32]. In this study, the heHb exhibited potent anti-staphylococcal activity, with an MIC of 3 μg mL^-1^, while its constituent, uliB, showed comparable inhibitory effects. These findings align with previous reports describing the antibacterial potential of heHb and uliB, which demonstrated activity within the same concentration range [12,13]. Moreover, other *Hypericum* species, such as *H. beanii*, *H. calycinum, H. foliosum, H. hircinum, H. lagarocladum, H. olympicum* and *H. revolutum*, have been reported to inhibit *S. aureus* at similar or higher concentrations (64 to 256 μg mL^-1^), supporting the genus as a rich source of bioactive metabolites [33]. The results obtained here further confirm the promising antibacterial profile of heHb and provide novel insights into its mechanism of action. By linking inhibitory activity with alterations in bacterial metabolism, oxidative stress induction and morphological damage, this work expands the current understanding of how heHb and uliB constituents act against resistant *S. aureus* strains, highlighting their potential as prototypes for new antimicrobial agents.

The inoculum effect, defined as the increase in MIC values with higher bacterial densities [34], is an important factor influencing antimicrobial efficacy. During infection, bacterial loads vary considerably across tissues and high-density infections, such as biofilms, abscesses or bloodstream, and chronic infections, pose major therapeutic challenges [35]. This phenomenon may result from reduced drug availability or induced bacterial tolerance [36]. In this context, both heHb and uliB maintained a consistent inhibitory effect even at elevated inoculum levels, suggesting that their antimicrobial activity is less affected by cell density, an advantageous property for treating infections associated with high bacterial loads.

SEM revealed that untreated *S. aureus* ATCC 29213 and USA300 cells exhibited intact and well-defined morphologies, whereas treatment with heHb and uliB caused pronounced surface alterations, including depressions, vesicle formation and roughened cell walls. Similar structural damage has been associated with the action of plant-derived antimicrobials such as citral and *trans*-cinnamaldehyde, which induced deformation and irregular cocci formation in *S. aureus* [37], and with extracts from *Phyllanthus emblica* and *Lycium shawii*, which caused extensive cell lysis and leakage of intracellular contents [38]. The rough and nodular cell surface observed after treatment with heHb and uliB resembles the morphological effects reported for rhamnolipid-treated *S. aureus* [39], which may reflect alterations in cell wall maintenance similar to those described previously, where *S. aureus* mutants lacking autolysin (*atl*) exhibited division defects and increased surface roughness [40].

TEM complemented these findings, showing loss of the characteristic spherical shape, septal malformation and cytoplasmic disorganization in treated cells. The alterations resembled those described for *S. aureus* exposed to *Hymenaea stigonocarpa* extracts, which disrupted cell division and induced membrane rupture [41]. Treatments with uliB led to additional structural changes, including increased cell wall thickness and surface depressions, similar to those observed in *S. aureus* mutants lacking *lcpC*, a gene essential for peptidoglycan assembly [42]. Together, these results suggest that heHb and its constituent uliB compromise the integrity of the *S. aureus* cell envelope, potentially by interfering with cell wall synthesis and remodelling.

Consistent with the observed morphological alterations, previous studies have shown that plant-derived compounds can affect *S. aureus* cell integrity through distinct mechanisms. For instance, Zeng *et al.* [43] and Dai *et al.* [44] reported extensive membrane permeabilization in *S. aureus* following treatment with *Polygonum chinense* and citral, with more than 60 to 90 % of cells staining positive for propidium iodide. In contrast, the low membrane labelling observed for heHb and uliB suggests a milder or indirect impact on the plasma membrane, indicating that their antimicrobial activity likely involves other cellular targets. In this sense, comparable effects on the metabolic activity of *S. aureus* have been reported for several plant-derived products that interfere with the bacterial electron transport chain. Al-Bakri and Afifi [45] observed that extracts rich in alkaloids, terpenoids and phenolics reduced cellular metabolism, suggesting that these secondary metabolites can impair bacterial energy production. The reduction in metabolic activity observed for heHb and uliB may therefore be associated with a similar mechanism, potentially involving interference with the electron transport chain or related metabolic pathways rather than direct damage to the cell membrane.

*S. aureus* possesses efficient defence systems against oxidative stress, mainly through catalase, superoxide dismutase and the carotenoid pigment staphyloxanthin, which confers antioxidant protection and contributes to bacterial survival during infection [20,46]. The induction of ROS observed after exposure to heHb extract and uliB suggests that these treatments may disrupt this protective balance, promoting oxidative damage. A similar mechanism has been described for bactericidal drugs, which trigger hydroxyl radical formation through the Fenton reaction and alter metabolic redox processes [47], supporting the hypothesis that oxidative stress plays a key role in the antimicrobial action of these compounds. Consistently, the partial restoration of bacterial growth in the presence of the antioxidant N-acetylcysteine observed in our study supports the involvement of ROS in the antimicrobial effect and indicates that oxidative stress is not the sole mechanism underlying the compounds' activity.

Biofilm-associated infections pose a major therapeutic challenge because the extracellular matrix and altered metabolic states reduce antimicrobial penetration and efficacy [48]. In our study, heHb and uliB both inhibited biofilm formation, yet sub-MIC exposure promoted biofilm induction. This response is consistent with adaptive mechanisms described under sub-inhibitory antimicrobial pressure, including increased exopolysaccharide synthesis and enhanced adhesiveness, and may also be linked to stress-response pathways triggered by metabolic imbalance and oxidative stress [49,50]. Importantly, mature biofilms were susceptible to disruption only at supra-MIC concentrations, consistent with literature showing that higher drug levels are generally required to disassemble established biofilm architecture [51]. These biofilm dynamics align with our additional findings: the compounds induced a pronounced, dose-independent suppression of metabolic activity and increased ROS production, while causing minimal membrane permeabilization. Together, this pattern supports a model in which interference with cellular energy metabolism and redox homeostasis, rather than primary membrane disruption, contributes to both the inhibition of biofilm formation, while ensuring sufficiently high drug concentrations to effectively disrupt established biofilms and the susceptibility of mature biofilms to higher drug pressure. Clinically, these results reinforce the need for dosing strategies that avoid prolonged sub-inhibitory exposure, which may promote biofilm formation, while achieving sufficiently high concentrations to disrupt established biofilms.

Given experimental evidence that uliB affects the energy metabolism of *S. aureus*, molecular docking was performed to explore its potential interaction with two key enzymes of the bacterial electron transport chain: NDH-2 and CydA. Since *S. aureus* relies on NDH-2 and cytochrome *bd* oxidase for respiration, both absent in mammalian cells, these enzymes represent selective targets for antimicrobial development [52,53]. The results indicated that uliB binds to NDH-2 in a mode like ubiquinone and the known inhibitor myricetin [54], suggesting competitive inhibition at the quinone-binding site. Likewise, the compound showed favourable binding to CydA, forming interactions comparable to those of reported inhibitors such as auraquine D [55], although with lower affinity than quinestrol. Taken together, these findings are consistent with NDH-2 as a putative molecular target of uliB and are in line with the experimental evidence of disrupted energy metabolism and reduced bacterial viability.

The toxicity of test compounds on *in vitro* and *in vivo* models was investigated. The haemolytic activity of heHb and uliB was evaluated since erythrocytes are a suitable model for initial cytotoxicity screening [56]. Both showed less than 10 % haemolysis after 24 h at 156.2 and 78.12 μg mL^-1^, respectively, indicating low toxicity, similar to findings for other plant extracts such as *Chamaemelum nobile* and *Mentha pulegium* [57]. Although plant metabolites can occasionally induce erythrocyte rupture [58], the heHb effect appeared to be mild and possibly time-dependent [59]. To complement these results, cytotoxicity was assessed in Vero and HaCaT cells. UliB showed higher CC₅₀ values than the extract (97.6 and 212.26 μg mL^-1^, respectively), and the SI indicated greater antibacterial selectivity toward *S. aureus* than cytotoxicity toward epithelial cells. These values exceeded those reported by Akinboye *et al.* [60] for *Erythrina caffra* (SI = 3.23-8.55) and by Fontanay *et al.* [61] for triterpenes with low SI (<10), reinforcing the safety and selectivity of both natural products. Considering the ethical principles of the 3Rs (replacement, reduction and refinement) in toxicity testing [62], *Galleria mellonella* larvae were used as an *in vivo* alternative model. Neither heHb (625 μg mL^-1^) nor uliB (312.5 μg mL^-1^) caused mortality within 168 h, supporting their low toxicity, in agreement with results for *A. colubrina* [63] and *R. officinalis* [64]. Together, these findings indicate that both the extract and uliB exhibit promising biological activity with minimal toxicity in *in vitro* mammalian cells and *in vivo* in *G. mellonella* larvae.

Given that pharmacokinetic limitations and toxicity issues are among the primary causes of drug development failure [65], the pharmacokinetic and toxicity profile of uliB was investigated *in silico* approaches as a preliminary assessment of its potential as an antimicrobial candidate. The predictions suggest that uliB may have properties consistent with oral bioavailability, which could be advantageous for treatment adherence and cost reduction [66], although these findings require experimental validation. Among the transporters analysed, P-glycoprotein (PgP), a mammalian efflux pump, plays a key role in modulating the pharmacokinetic properties of many compounds, including antibiotics. PgP can limit drug accumulation at the target site by decreasing cellular availability and exposure time, thereby reducing antimicrobial efficacy [67]. Based on *in silico* predictions, uliB may interact with PgP; however, the functional relevance of this interaction remains to be experimentally determined. Additionally, considering that mammalian and bacterial efflux pumps may share substrates, and that some natural products exhibit dual inhibitory activity, uliB may potentially display similar behaviour. Nevertheless, this hypothesis remains speculative and warrants further investigation, particularly in the context of neuroinfections.

## Conclusions

The extract of *H. brasiliense* and its isolated compound, uliginosin B, exhibited comparable inhibitory effects against various *S. aureus* strains, warranting further investigation into their antimicrobial activity. Both samples inhibited sessile growth and affected biofilm dynamics, while inducing oxidative stress and reducing bacterial metabolic activity, even at high cell densities. These findings suggest that their antibacterial effects may be associated with interference in cellular energy metabolism and redox balance. Although *in silico* analyses and phenotypic assays suggest a possible interaction between uliginosin B and NADH dehydrogenase (NDH-2), this mechanism remains hypothetical and requires direct enzymatic validation. The selectivity index indicates preferential activity against *S. aureus* compared to mammalian cells, while *in silico* pharmacokinetic predictions suggest that uliginosin B may present drug-like properties. However, these results should be interpreted as preliminary and require experimental confirmation. Importantly, this study has some limitations, including the absence of direct target validation and reliance on predictive models for pharmacokinetic assessment. Despite these limitations, the results support the potential of *H. brasiliense* hexane extract and uliginosin B as promising leads to the development of new antimicrobial strategies against drug-resistant *S. aureus*, warranting further mechanistic and *in vivo* investigations.
